# Nanooptomechanical Transduction in a Single Crystal with 100% Photoconversion

**DOI:** 10.1021/acs.jpcc.1c02457

**Published:** 2021-04-19

**Authors:** Jacqueline M. Cole, David J. Gosztola, Jose de J. Velazquez-Garcia

**Affiliations:** † Cavendish Laboratory, Department of Physics, University of Cambridge, J.J. Thomson Avenue, Cambridge CB3 0HE, U.K.; ‡ ISIS Neutron and Muon Source, STFC Rutherford Appleton Laboratory, Harwell Science and Innovation Campus, Didcot OX11 0QX, U.K.; § Department of Chemical Engineering and Biotechnology, University of Cambridge, West Cambridge Site, Philippa Fawcett Drive, Cambridge CB3 0AS, U.K.; ∥ Argonne National Laboratory, 9700 South Cass Avenue, Lemont, Illinois 60439, United States

## Abstract

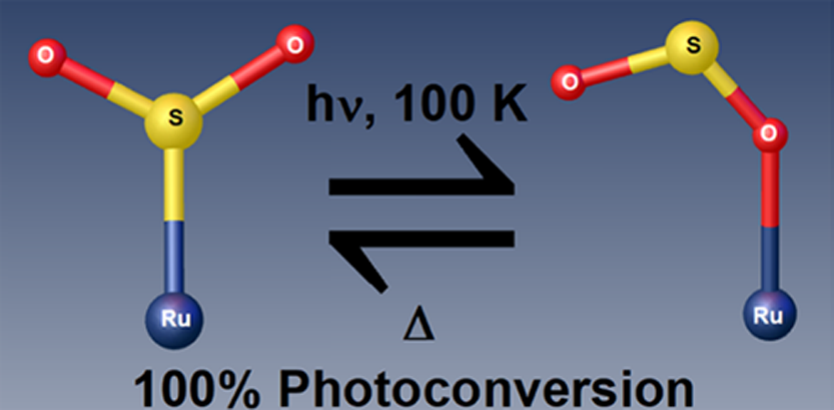

Materials that exhibit nanooptomechanical transduction in their single-crystal form have prospective use in light-driven molecular machinery, nanotechnology, and quantum computing. Linkage photoisomerization is typically the source of such transduction in coordination complexes, although the isomers tend to undergo only partial photoconversion. We present a nanooptomechanical transducer, *trans*-[Ru(SO_2_)(NH_3_)_4_(3-bromopyridine)]tosylate_2_, whose S-bound η^1^-SO_2_ isomer fully converts into an O-bound η^1^-OSO photoisomer that is metastable while kept at 100 K. Its 100% photoconversion is confirmed structurally via photocrystallography, while single-crystal optical absorption and Raman spectroscopies reveal its metal-to-ligand charge-transfer and temperature-dependent characteristics. This perfect optical switching affords the material good prospects for nanooptomechanical transduction with single-photon control.

## Introduction

Materials that undergo single-crystal optical actuation are gaining traction as an emerging research field for materials chemistry, given their attractive solid-state optical switching and transduction capabilities.^1−5^ Prospective applications range from optical sensing^6^ to light-driven molecular rotors,^7^ to photocatalysis,^8^ to futuristic circuitry for quantum computers.^9^ Their single-crystal form provides a high-quality solid-state medium for single-photon control. Coordination complexes that exhibit single-crystal optical actuation via linkage photoisomerization are of particular interest since their metal core offers them good thermal stability, which is needed for prospective photonic applications.

Linkage photoisomers have been identified in a range of coordination complexes,^10,11^ especially via the technical advances of photocrystallography,^12−15^ which afford light-induced crystal structures. “Seeing is believing”, whereby N_2_, NO, NO_2_, and SO_2_ linkage photoisomerization in Group 8 and Group 10 complexes have dominated photocrystallographic results thus far.^10,11,16−31^ Nonetheless, the linkage isomer does not completely photoconvert in all but a few^32^ of these light-induced crystal structures; rather, one or more photoisomers tend to form within a predominantly dark-state crystal structure environment. As such, these photoisomers have to be modeled as a minor disordered component of the dark-state isomer within its crystal-lattice ensemble.

All of these photocrystallographic results are examples of crystalline optical switches. Yet, few of them can also act as nanooptomechanical transducers, i.e., a light-driven switching process in which one molecule or ion stimulates mechanical motion in a neighboring molecule or ion. Certain members of a ruthenium sulfur dioxide-based (hereafter [RuSO_2_]) family of complexes offer some of these rare exceptions.^26−28^ This series of compounds is based on the generic formula, *trans*-[Ru(SO_2_)(NH_3_)_4_X]^*m*+^Y*_n_*, whose ligand, X, lies *trans* to the SO_2_ group that manifests solid-state linkage photoisomerization, while Y is a counter ion; *m* and *n* are integers that simply depict charge-balancing requirements, depending on the nature of X and Y. Only certain combinations of X and Y will afford nanooptomechanical transduction, the first example of which was reported by Sylvester and Cole, where X = 3-chloropyridine and Y*_n_* = tosylate_2_ or chlorobenzenesulfonate_2_.^26^ For each light-responsive cation, an S-bound η^1^-SO_2_ ligand photoisomerizes into an O-bound η^1^-OSO photoisomer with the minor component of an η^2^-(OS)O photoisomer also forming. The terminal oxygen of the η^1^-OSO photoisomer points toward the arene ring of one of the anions, to which it is so close that the arene ring rotates to mitigate crystal-lattice strain. Thus, a small mechanical change (SO_2_ photoisomerization in the cation) photostimulates a much larger mechanical motion (the arene ring in the anion). This work led to the discovery of a few more instances of nanooptomechanical transduction in this family of complexes.^27,28^ However, none of them exhibited a higher η^1^-OSO photoconversion fraction than was achieved in the first discovered example (36% of η^1^-OSO photoisomers).^26^ This limitation in optical switching thus limited the extent of nanooptomechanical transduction that could occur.

This work reports the discovery of *trans*-[Ru(SO_2_)(NH_3_)_4_(3-bromopyridine)]tosylate_2_ (**1**), which exhibits nanooptomechanical transduction with 100% conversion of dark-state SO_2_ ligands to their η^1^-OSO photoisomeric configuration. Its perfect switching capability could be especially useful for solid-state optical applications, where clean switching tends to be of paramount importance.

## Experimental Methods

**1** was synthesized from *trans*-[Ru(SO_2_)(NH_3_)_4_Cl]Cl, which was synthesized according to a literature procedure.^33^ Five milligrams (16 μmol) of this precursor and 5 μL (86 μmol) of 3-bromopyridine were dissolved in 1 mL of water, to which a solution of *p*-tosylic acid (200 μL, 2 M; >98% purity, Sigma-Aldrich) was added dropwise. This produced a precipitate of yellowy-orange platelike crystals after 2–4 h, which were isolated through vacuum filtration and washed three times with methanol. Dark and light-induced structures of **1** were characterized by photocrystallography at 100 K. 500 nm LED light (ThorLabs M505F3) was used to photostimulate the crystal. Single-crystal optical absorption spectroscopy was used to determine the metal-to-ligand charge-transfer (MLCT) characteristics and thermal stability of **1**, using the 505 nm LED light as the optical pump; spectra were acquired at 100, 133.5, and 215 K. The latter two values correspond to temperatures where the SO_2_ in **1** is expected to thermally decay into the side-bound η^2^-(OS)O photoisomer or the η^1^-SO_2_ dark-state isomer, based on results from known nanooptomechanical transducers in the [RuSO_2_] family of complexes.^26−28^ Single-crystal Raman spectroscopy on **1** characterized its molecular structure and thermal stability in more detail. Detailed experimental procedures for these three *in situ* light-induced single-crystal characterization methods are given by Cole et al.,^31^ while further details specific to **1** are given in the Supporting Information.

## Results and Discussion

### Single-Crystal Nanooptomechanical Transduction Mechanism in **1**

**Figure 1:**
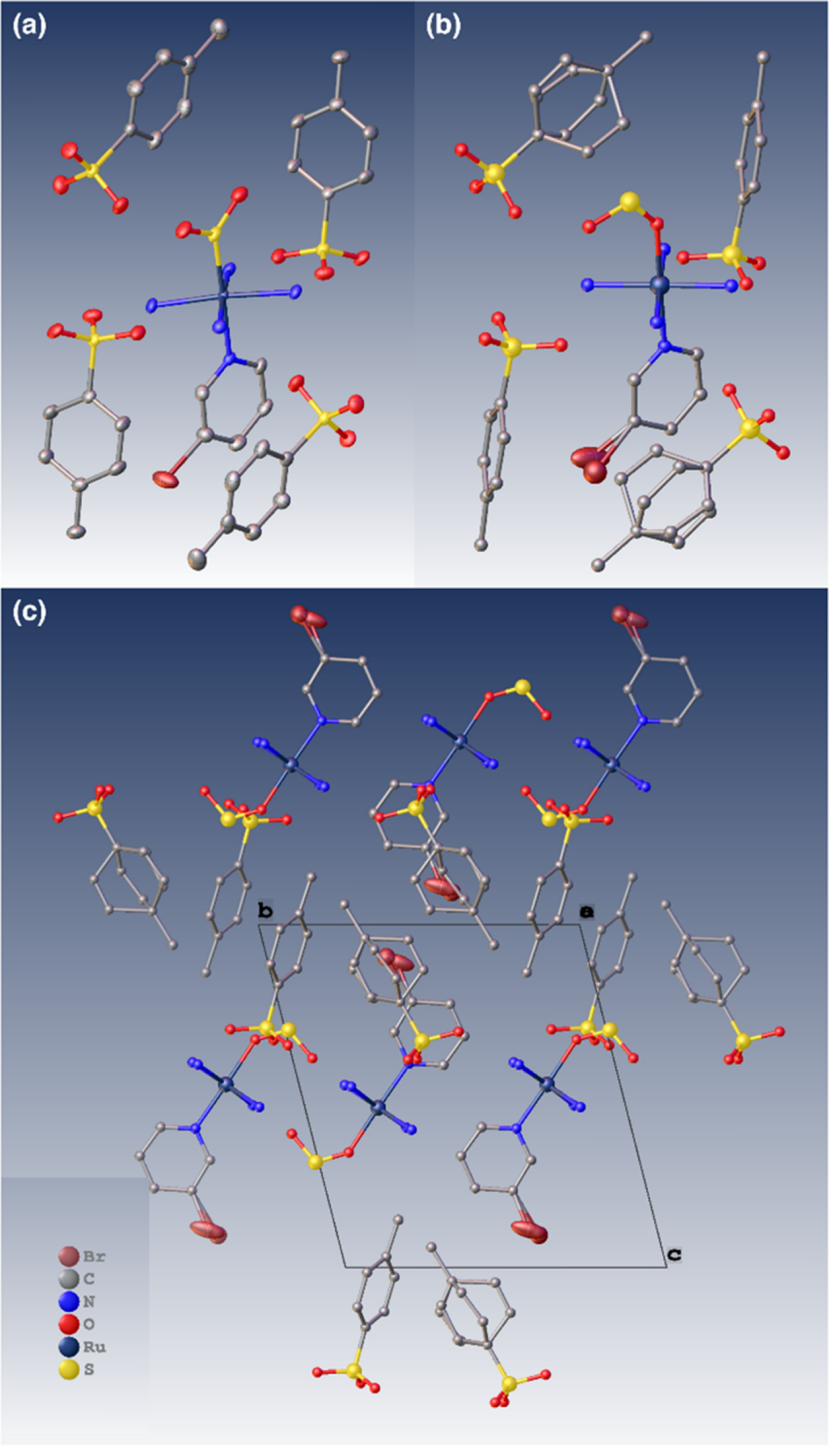
(a) Dark and (b) light-induced crystal structure of **1**, showing the SO_2_ isomer surrounded by a reaction cavity of tosylate anions. (c) Crystal packing of **1** looking down the crystallographic *a*-axis. The disorder in Br is shown explicitly and with ADPs to indicate its role in interionic interactions.

The dark-state and light-induced crystal structures of **1** are displayed in Figure 1. The 100% η^1^-SO_2_ to η^1^-OSO photoconversion observed in **1** is unprecedented in any [RuSO_2_] complex. Moreover, a fully formed η^1^-OSO isomer has never been reported, at least crystallographically, in any Ru-based organometallic complex; and there are only 11 instances of fully formed η^1^-OSO–metal coordination for any metal, within the Cambridge Structural Database:^34^ six instances across Ti, Mn, Co, Ni, and Zn first-row metals^35−39^ and five instances of one second-row metal, Ag.^40−44^

**Scheme 1:**
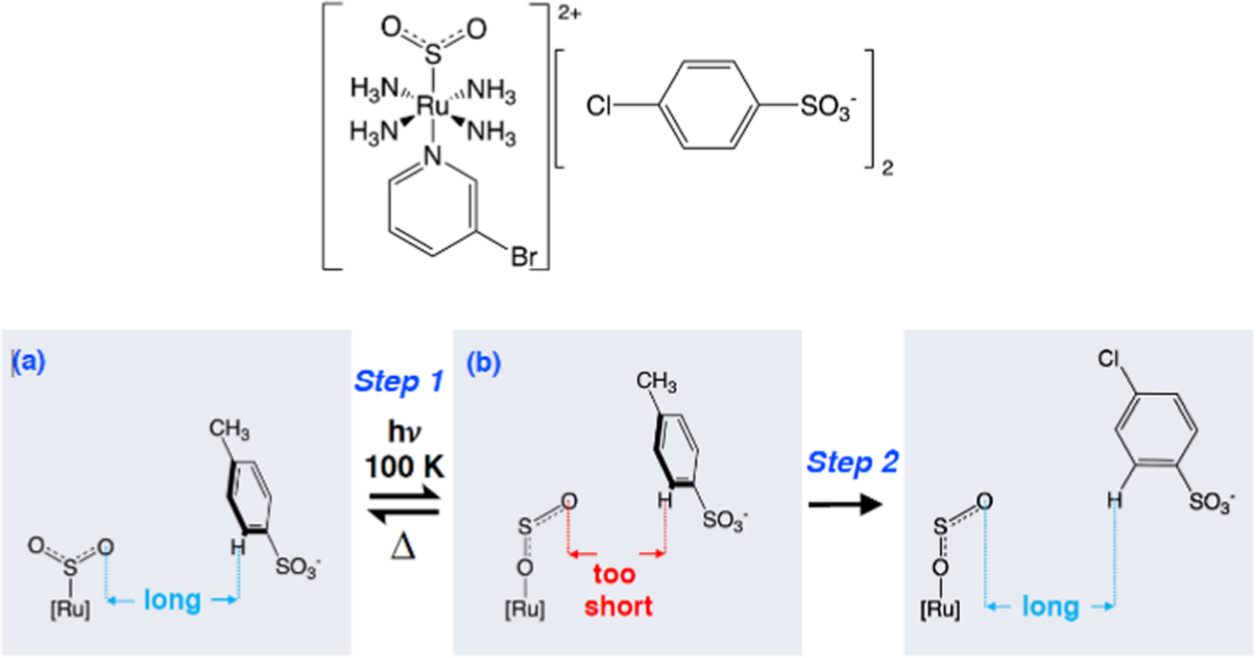
Operational Mechanism of Nanooptomechanical Transduction for [RuSO_2_] Complexes Proposed by Sylvester and Cole^26^

In practical terms, **1** not only behaves as a perfect optical switch, it is also a nanooptomechanical transducer. The light-induced crystal structure determination (see the Supporting Information) reveals that 47.1(9)% of the arene rings have twisted in the tosylate anion that lies closest to the halogen substituent of the cation within the crystallographic asymmetric unit of **1** (hereafter called the “rotor ring”). The rotor ring has twisted by 68(3)° in response to the 100% η^1^-OSO formation, which is markedly higher than that of its previously determined 3-chloropyridine analogue, *trans*-[Ru(SO_2_)(NH_3_)_4_(3-chloropyridine)]tosylate_2_ (39°).^26^ The overall ratio of the photoconversion fraction for the η^1^-OSO photoisomer: twisted rotor rings in **1** (100:47.1 = 2.1) is in line with that of this 3-chloro analogue (42:22 = 1.9).^26^ Given this approximate 2:1 ratio, and the 100% η^1^-OSO formation, it would seem that a maximum of 50% of the rotor rings will twist in response to η^1^-OSO formation. One might have expected a 1:1 rather than this observed 2:1 correspondence pending that the arene rotation occurs as a direct mechanical response to the η^1^-OSO formation. Indeed, this lack of one-to-one correspondence was deemed curious for the 3-chloro analogue.^26^ This raises suspicion that the operational mechanism behind this transduction process is more complicated than was first envisaged (Scheme 1).

**Figure 2:**
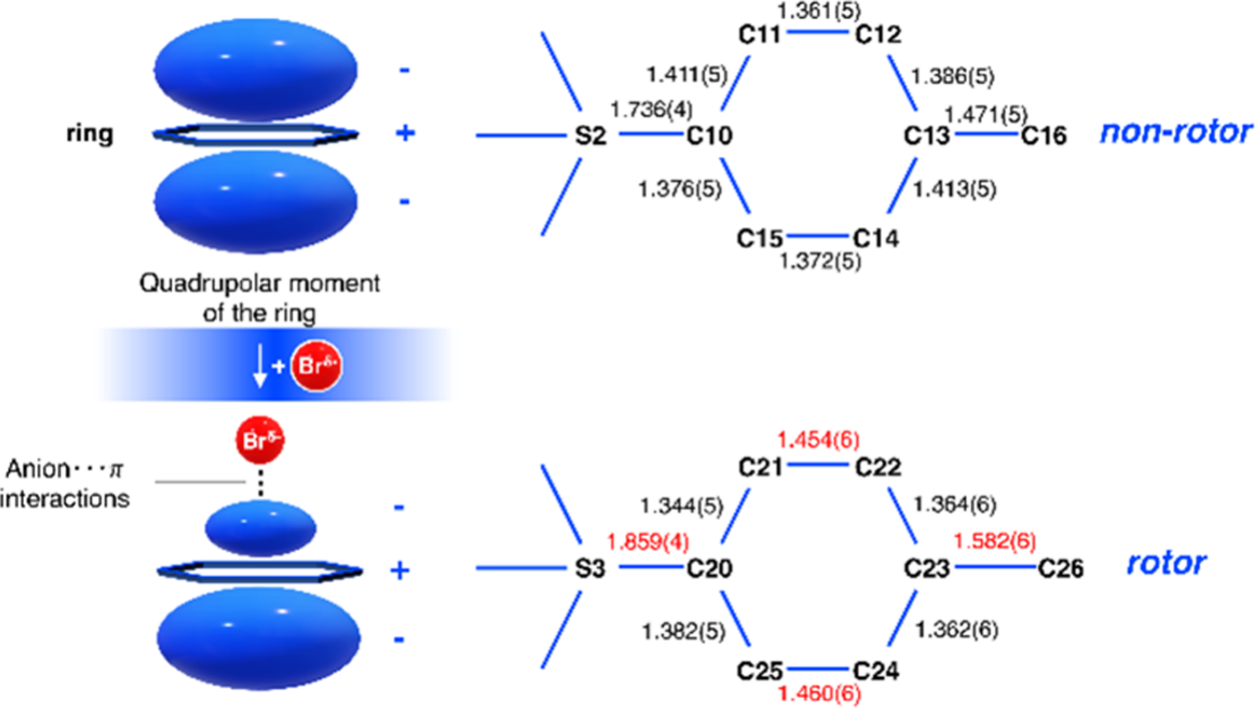
(Left) Quadrupolar moment of a ring that engages with nucleophiles to form anion···π interactions. (Right) Selected bond lengths of the two crystallographically independent tosylate ions in **1**; those in red denote the antiquinoidal bonding pattern that is present in the rotor ring, which gives rise to its highly polarizable form making it susceptible to engage in anion···π interactions.

The light-induced crystal structure of **1** could only be suitably modeled with the anisotropic displacement parameters (ADPs) of the pyridyl carbon atoms showing significant libration; this suggests that this entire ligand is weakly bound. Moreover, its 3-bromo substituent could only be refined sensibly when it was modeled with 50:50 positional disorder. At first sight, this is counterintuitive given that the disordered bromine atoms lie out of the plane with bond lengths which are shorter and longer than the expected value for a C_ar_–Br bond, cf. 1.689 Å (C2–Br1) and 2.034 Å (C2–Br1A) in **1** compared with the average C_ar_–Br bond length of 1.899 Å.^45^ Yet, attempts to model the Br substituent with a single ADP instead of positional disorder left far too much unmodeled electron density (see the Supporting Information). In fact, Br could only be modeled satisfactorily with two distinct ADP contributions, whereby the ADP of Br1 is highly elliptical, extending toward the nonrotor ring of the tosylate anion, whose carbon ADPs align in the same direction as Br1. The ADP of Br1A is essentially sandwiched between the two parallel planes of the pyridine ring and the rotor ring. The ADP of Br1A has a more spherical form. This need to model Br with two distinct ADPs, the position, direction, and magnitude of the ADPs of Br relative to their neighboring tosylate anions, the nucleophilic nature of bromine, the photolabile nature of C–Br bonds,^46^ and the close proximity of Br1A to the rotor ring (see the Supporting Information) are all indicative of anion···π interactions existing between the Br and the tosylate ion. Such interactions are now recognized and have been shown to be energetically favorable, typically ranging from 20 to 50 kJ/mol.^47−51^ This energy range is precisely in line with the typical activation energy, *E*_a_, of thermally stimulated η^1^-OSO to η^2^-(OS)O reverse isomerization that is seen in these [RuSO_2_] complexes.^52^ So the formation of anion···π interactions in a [RuSO_2_] complex would seem to help stabilize the η^1^-OSO photoisomer from thermal decay. The more spherical ADP for Br1A is indicative of it forming defined anion···π interactions with the rotor ring (see Figure 2). The strongly antiquinoidal (highly polarizable) character of the rotor ring, as observed via its bonding patterns (see Figure 2), also evidences a favorable environment for the rotor ring to form anion···π interactions, in stark contrast to the other crystallographically independent tosylate ion (the nonrotor) whose ring bonding pattern is distinctly uniform (Figure 2). The fact that the rotor ring undergoes nanooptomechanical transduction is also consistent with the energy-stabilizing nature of anion···π interactions that draw in Br1A, whereby the ring atoms in the tosylate ion rotate to best accommodate this nucleophile. The average separation between Br1A···H–C_ar_ interactions increases once the rotor ring transduces (see Table S1), evidencing such stabilization.

In contrast, the parallel alignment of the heavily elongated ADP for Br1 with the carbon ADPs of the other crystallographically independent tosylate ion (the nonrotor ring) suggests that it is trying unsuccessfully to form anion···π interactions: Br1 is not so distant from the nonrotor ring (see Table S1), but the ring is not sufficiently polarizable to engage in such interactions (see Figure 2).

The C–Br bond is thus heavily distorted as a result of competing demands on the Br atom, which is not so surprising when considering that it also has to contend with its photolabile nature. The C–Br bond does not cleave, presumably because it is surrounded by a crystal-lattice medium whose forces contain it well. Moreover, the contrasting roles for the Br1 and Br1A components of Br counter each other in direction, bringing a net restorative force between them.

While it is clear that the η^1^-OSO formation in **1** stimulates the arene ring rotation, the precise mechanism of transduction needs revisiting, given the evidence for these anion···π interactions as well as the uneven (2:1) ratio of % η^1^-OSO:% rotor ring formation formed upon photolysis, in contrast to the 1:1 ratio (50:50) of disorder that is common to bromine and the rotor ring in **1**. Moreover, the protruding oxygen atom of the η^1^-OSO photoisomer in **1** is close to the rotor ring, but it is directed toward the side of ring, while Br1A lies above the rotor ring and projects toward the ring to form a direct Br1A^δ−^···π interaction.

Given these observations, we now consider the viability of a more indirect mechanism for transduction than was previously supposed.^26^Table 1 shows that the Ru–N_ammine_ bonds involving the equatorial ammine ligands all contract significantly upon η^1^-OSO formation; this is unusual as the strength of ammine ligand coordination in [RuSO_2_] complexes tends not to change considerably upon SO_2_ linkage photoisomerization.^28,30^ The Ru–O and Ru–N bonds in **1** that involve the two *trans* ligands both extend upon light activation (see Table 1). Thus, the linkage photoisomer and the 3-bromopyridine ring are both more weakly coordinated to the Ru ion compared with the dark-state configuration of **1**. There will be a *trans* influence of the 3-bromopyridine ligand in **1** owing to the S-bound η^1^-SO_2_ to O-bound η^1^-OSO photoconversion. Meanwhile, the bromopyridine ring will become more labile, as is evidenced via the significant libration observed in all C and Br atoms of this ligand in the light-induced crystal structure of **1**. This will encourage an already innate tendency of the C–Br bond toward photolability. Yet, the C–Br bond does not cleave, presumably because Br is held by the crystal-lattice forces of its surrounding environment. Nonetheless, 50% of Br becomes close enough to the rotor ring that it forms anion···π interactions and this, in turn, stimulates mechanical transduction in a corresponding 50% of the rotor rings.

**Table 1:** Bond Lengths of the Ru Coordination Environment for the Dark-State and Light-Induced Crystal Structures of **1**

bond	bond length (dark), Å	bond length (light), Å
Ru1–N1_(ammine)_	2.168(3)	2.119(6)
Ru1–N2_(ammine)_	2.197(3)	2.125(6)
Ru1–N3_(ammine)_	2.182(3)	2.129(6)
Ru1–N4_(ammine)_	2.195(3)	2.118(6)
Ru1–N5_(3-Brpyridyl)_	2.042(3)	2.075(6)
Ru1–S1/O1_(SO_2_)_	2.0344(9)	2.063(5)

**Scheme 2:**
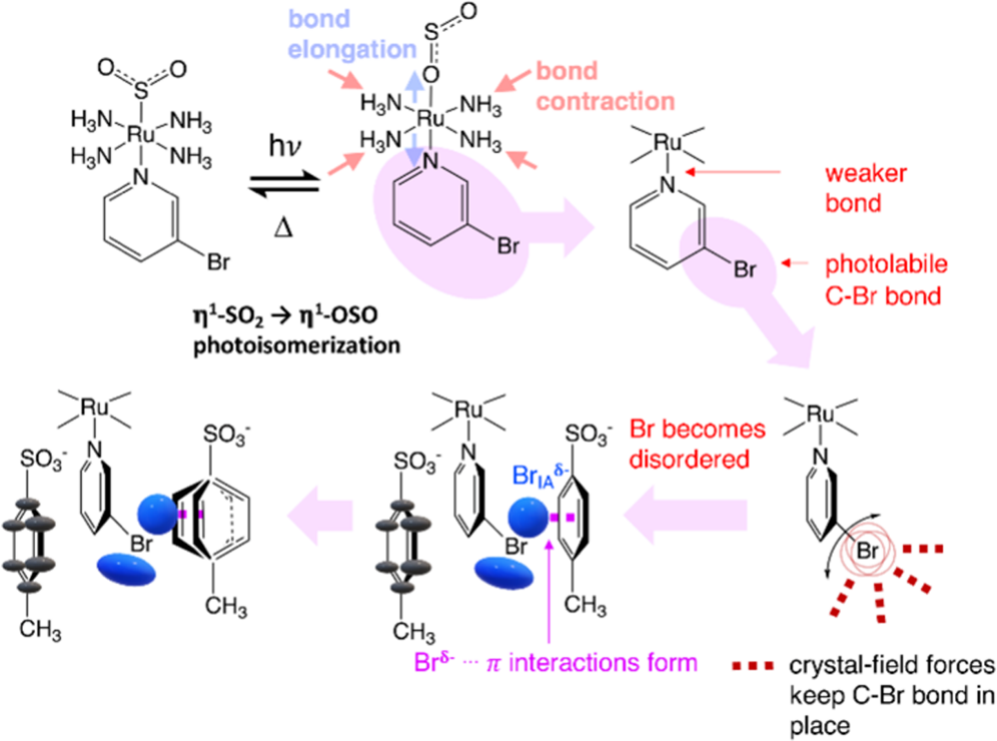
Overall Operational Mechanism for Nanooptomechanical Transduction Deduced for **1**

Overall, it would thus seem that nanooptomechanical transduction occurs via a knock-on effect of light forming the η^1^-OSO isomer that weakens the coordination of the 3-bromopyridine ligand to the Ru ion which, in turn, encourages its C–Br bond toward photolability, which is contained by the crystal-lattice environment but nonetheless forms anion···π interactions in a tosylate ion whose ring rotates to accommodate these interactions. Scheme 2 illustrates this deduced mechanism for nanooptomechanical transduction in **1**.

The η^1^-OSO interactions with the other side of the rotor ring that were previously identified in the 3-chloropyridyl analogue of **1**(26) will nonetheless encourage the transduction, but it would appear that this is not the primary cause of transduction, at least in **1**. We prospect that the 3-chloropyridine analogue of **1** operates via the same mechanism as **1**, since this analogue crystallizes in an isomorphous unit cell framework, and it evidences significant residual electron density in the chlorine atoms of its 3-chloropyridine ligand in the direction perpendicular to its pyridyl ring, i.e., akin to that of the Br in **1** (see Figure S2 in the Supporting Information of ref (26)). Nonetheless, one needs to be careful not to make a direct comparison owing to the different light source used in that work.

The light-induced crystal structure of **1** was determined twice to confirm the verity of the results that led to this newly proposed mechanism. All relevant structural characteristics were found to be highly reproducible (see the Supporting Information).

One thing that remains uncertain is why **1** yields 100% photoconversion to its η^1^-OSO configuration at 100 K, as this contrasts with the partial photoconversion levels that have been witnessed in all other [RuSO_2_] complexes.^20−31^ This is particularly intriguing since some of those complexes also behave as transducers and carry the same tosylate counterions, and crystallize with similar unit cell parameters.^26,27^ The most evident structural difference between **1** and all of these other complexes is the anomalous disordered behavior of the Br substituent. Indeed, we have already seen that this influences the operational mechanism of nanooptomechanical transduction in **1**. Thus, the most logical deduction is that the disordered behavior of the Br atom relates to this 100% photoconversion, given the information available. Its disordered nature will presumably influence the photoreaction cavity of the SO_2_ isomer in some way.^23^ However, further work is needed to verify such a proposition and understand the nature of this relationship, should this turn out to be the case.

### Metal-to-Ligand Charge-Transfer Characteristics of **1**

**Figure 3:**
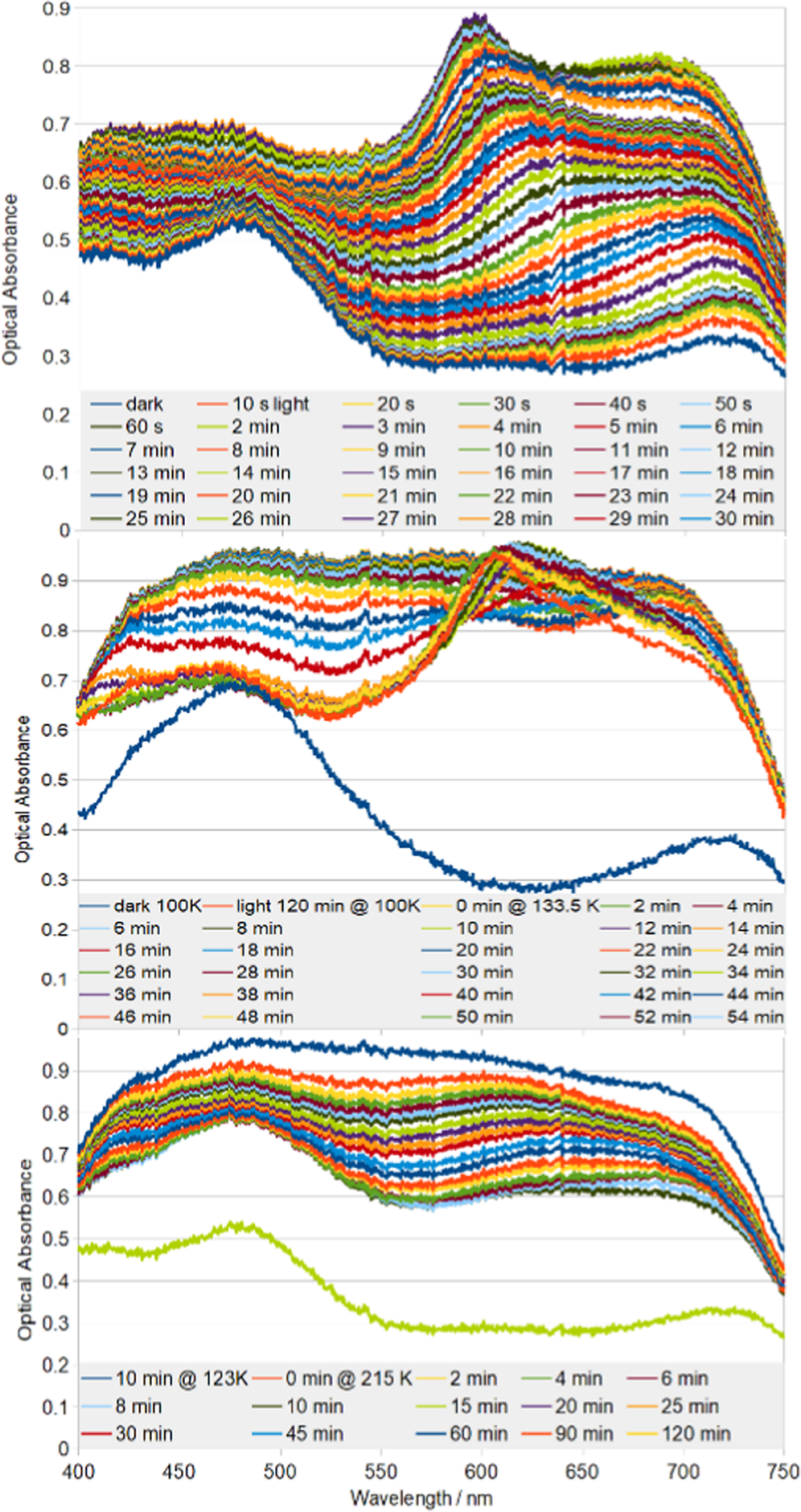
Single-crystal optical absorption spectra of **1** (top) at 100 K while being exposed to 505 nm light for time, *t*; (middle) after *t* min of being held at 133.5 K having been raised to this temperature from its 120 min light-exposed state at 100 K, (bottom) after *t* min of being held at 215 K having been raised to this temperature from its 120 min light-exposed state at 123 K.

The 100% η^1^-SO_2_ to η^1^-OSO photoconversion in **1** means that metal-to-ligand charge-transfer (MLCT) characteristics which are associated with its η^1^-OSO photoisomer can be isolated from any other SO_2_ isomeric contribution. Figure 3 (top) shows the single-crystal optical absorption spectrum of **1** as it is progressively exposed to 505 nm light. The absorption rises steadily across the full panchromatic range of visible light. Initially, **1** absorbs more light in the red region of the spectrum. The peak so formed starts to undergo a hypsochromic shift after ca. 10 min of light exposure, which culminates in an absorption peak maximum forming at ca. 600 nm. All of these absorption features fall under a broad envelope of MLCT that spans ca. 525–750 nm. Optical absorption is less rapid at lower wavelengths (λ < 525 nm), which evidences a distinct band of MLCT in that region.

The formation of a broad envelope of MLCT in the ca. 525–750 nm region of optical absorption is characteristic of η^1^-OSO formation in this family of [RuSO_2_] complexes.^28,30^ The hypsochromic shift observed within this MLCT band of **1** is more curious, as it is absent in *trans*-[Ru(SO_2_)(NH_3_)_4_Cl]chlorobenzenesulfonate_2_ which also acts as a nanooptomechanical transducer upon 26.0(3)% η^1^-OSO formation,^28^ while it is present in *trans*-[Ru(SO_2_)(NH_3_)_4_(3-phenylpyridine)]Cl_2_ which exhibits 52(3)% η^1^-OSO photoconversion.^30^ The absorption peak in the latter also lies at ca. 600 nm. Notwithstanding the anions, none of which would absorb within the visible range of light, the pyridyl-based ligand is the distinguishing chemical component that will undergo MLCT and is common to both [RuSO_2_] complexes whose optical absorption peaks at ca. 600 nm following a hypsochromic shift, but is absent in *trans*-[Ru(SO_2_)(NH_3_)_4_Cl]chlorobenzenesulfonate_2_. Accordingly, we assign this hypsochromic shifting and peak maximum in **1** to MLCT processes that are associated with the pyridyl ligand. This shifting stands to reason given that the Ru will coordinate more weakly to the η^1^-OSO photoisomer than to its S-bound SO_2_ isomer.^53^ Thus, the progressive O-bound η^1^-OSO formation will weaken the Ru coordinative environment whose strength needs to be recovered by other ligands. The equatorial ammine ligands in **1** contribute significantly to this recovery, as witnessed by the significant Ru–N_ammine_ bond contractions that occur between the dark and light-induced crystal structure (see Table 1). As discussed earlier, this observation is unusual given that Ru–ammine ligand bond lengths remain similar when exposed to light in other [RuSO_2_] complexes that form η^1^-OSO photoisomers.^28,30^ The 100% level of η^1^-OSO photoconversion in **1** may partly explain this observation. However, the ammine ligand involvement may also be needed since the 3-bromopyridine ligand actually coordinates with Ru more weakly in its light-induced crystal structure (see Table 1). This counters the situation in the two aforementioned η^1^-OSO photoisomer-forming complexes, where the *trans* ligand to η^1^-OSO either maintains or decreases its coordinative strength to Ru.^28,30^ The photolability of the C–Br bond appears to disrupt the pyridyl ring in **1**, judging from the high levels of libration observed in the 3-bromopyridine ligand of the light-induced crystal structure. The extent of conjugation within this ligand will diminish as the libration increases and as the photolability of C–Br becomes more accentuated. Hence, the progressively hypsochromic nature of the shift observed in the optical absorption peak. These knock-on effects of the η^1^-OSO formation on the 3-bromopyridine ligand behavior are also consistent with the indirect nature of the MLCT characteristics that would be associated with nanooptomechanical transduction via the proposed mechanism for **1**.

The MLCT observed in the lower-wavelength (λ < 525 nm) region of the optical absorption spectrum for **1** presumably occurs because of the complete loss of dark state in **1**, i.e., 100% η^1^-OSO photoconversion. This assignment is presupposed since there is little absorption in this region for *trans*-[Ru(SO_2_)(NH_3_)_4_Cl]chlorobenzenesulfonate_2_, which exhibits only 26.0(3)% η^1^-OSO photoconversion,^28^ while more absorption is noted in this region for *trans*-[Ru(SO_2_)(NH_3_)_4_(3-phenylpyridine)]Cl_2_, which affords 52(3)% η^1^-OSO photoconversion.^30^ Even more absorption is observed in the light-induced form of **1** within this wavelength region. Thereby, the extent of optical absorption broadly matches these differences in η^1^-OSO formation. Note that this MLCT band is not thought to reflect a pyridyl contribution because only one MLCT band is present in similar compounds that have been measured previously, albeit in solution. For example, the [Ru(NH_3_)_4_(pyridyl)_2_]^2+^ ion in aqueous solution displays only one MLCT band,^54^ while this lies at 422 nm, one would expect this absorption band to be shifted by several hundred nanometers when measured in the crystalline state; indeed, such a shift was observed in another Group 8 transition-metal ammine complex when measured in these two surrounding media.^19^ We have already assigned one MLCT band to the pyridyl ligand in this work. Thus, this lower-wavelength (λ < 525 nm) MLCT band in **1** is assigned to a η^1^-OSO contribution, the extent of which seems to be broadly indicative of its photoconversion fraction.

### Thermal Stability of SO_2_ Linkage Isomers in **1**

The η^1^-OSO photoisomer is known to be thermally stable up to around 120 K, where it exists in previously reported [RuSO_2_] complexes.^26,27^ Such complexes thermally decay into the η^2^-(OS)O photoisomer above this temperature. **1** exhibits similar thermal-decay characteristics as can be seen in Figure 3 (middle). Thereby, the optical absorption of **1** progressively becomes a “black body” absorber once raised and held at 133.5 K over the course of a 3 h period. This black body absorption profile is characteristic of MLCT owing to the η^2^-(OS)O photoisomer in [RuSO_2_] complexes.^31,52^ The lower-wavelength (λ < 525 nm) region of the spectrum for **1** rises as the η^2^-(OS)O forms, corroborating our assignment of MLCT in this region to η^1^-OSO-Ru photoisomeric contributions since this decays during this process. The peak maximum that we assigned to a pyridyl contribution to MLCT at 100 K undergoes a bathochromic shift at 133.5 K, before blurring into the black body absorption profile as it forms. This observation also corroborates our Ru-pyridyl MLCT assignment at 100 K and its associated role in our proposed nanooptomechanical transduction mechanism for **1**, since **1** can no longer act as a transducer once the η^1^-OSO photoisomer has decayed; the η^2^-(OS)O photoisomer can only behave as an optical switch. Some dark-state η^1^-SO_2_ isomer may also coexist with the η^2^-(OS)O species, although this will be minor if present since the extent of optical absorption in the blue region of the spectrum of **1** lies well above that of the characteristic signature for the dark-state η^1^-SO_2_ configuration, shown in Figure 3 (middle) for reference.

The η^2^-(OS)O photoisomer in [RuSO_2_] complexes is known to revert entirely into a dark-state η^1^-SO_2_ configuration above ca. 200 K.^24,26−28^**1** follows this trend, as is evidenced by the panchromatic collapse of its optical absorption spectrum into the dark-state absorption profile once the temperature was raised and held at 215 K for a period of 5 h, as shown in Figure 3 (bottom).

**Figure 4:**
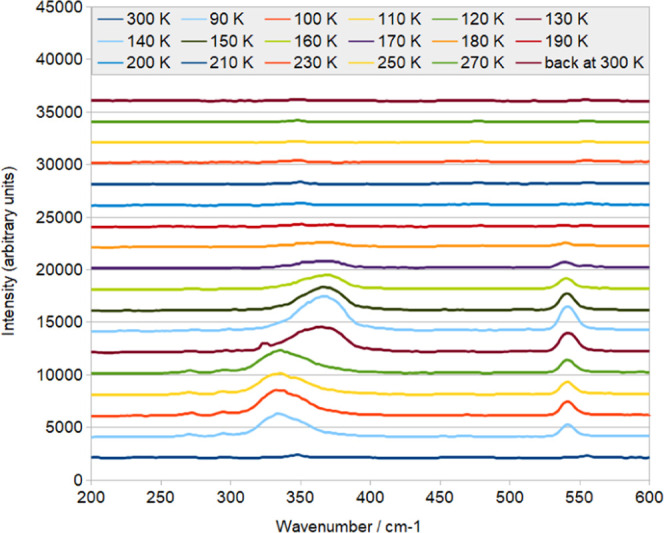
Multitemperature single-crystal Raman spectra of **1**, pumped and probed by 514.5 nm light at 90–300 K.

The thermal stability of **1** was further probed using single-crystal Raman spectroscopy with concerted optical microscopy, with data being acquired from 90 to 300 K. The Raman spectral features (Figure 4) remained essentially constant between 90 and 120 K but started to change at 130 K. By 140 K, the Ru–OSO vibrational stretch at 340 cm^–1^ had shifted to a considerably more intense peak at 360 cm^–1^, which is indicative of Ru-(OS)O vibrational stretch. The deformation mode of SO_2_ also became more intense by 140 K. The temperature range at which these Raman spectral changes occur (130–140 K) is consistent with the blue-to-yellow photochromic changes that are seen in the concerted optical microscopy of the crystal of **1**, which onsets at 140 K (see the Supporting Information). This range also mirrors that where the optical absorption spectra change as the η^1^-OSO photoisomer thermally decays. The intensity of both of these vibrational modes then diminishes as the temperature of the crystal is further elevated, becoming the same as the spectra at 300 K by about 200–220 K. This temperature range is where the η^2^-(OS)O photoisomer decays fully into the dark-state SO_2_ configuration, where a very weak Ru–S vibrational stretch is observed at 350 cm^–1^.

The Raman spectral characteristics for **1** are particularly useful for making unambiguous assignments of Ru-SO_2_, Ru–OSO, and Ru–(OS)O vibrational stretches in [RuSO_2_] complexes. This is because these vibrational modes are isolated from each other, owing to the 100% η^1^-SO_2_ to η^1^-OSO photoconversion in **1** and thence its thermal decay to η^2^-(OS)O at 130–140 K, which in turn reverts entirely to the η^1^-SO_2_ configuration at 200–220 K.

## Conclusions

In summary, we have discovered 100% η^1^-SO_2_ to η^1^-OSO linkage photoisomerism in a ruthenium sulfur dioxide complex, **1**, which acts as a nanooptomechanical transducer. The operational mechanism for this form of single-crystal optical actuation in **1** has been deduced by the photocrystallographic findings of this work and corroborated by single-crystal optical absorption spectroscopy. This mechanism tracks a more intricate mechanism than has been previously supposed for other [RuSO_2_] complexes.^26^ The formation of the η^1^-OSO photoisomer is still the stimulus for transduction, but the process is more indirect than previously supposed, with MLCT modulations relaying through the cation causing knock-on effects that induce the transduction. To this end, the 1:1 correspondence between the extent of disorder in the bromine atom and an arene ring in **1** is rationalized. The photolabile Br^δ-^ constituent of **1** appears to interact with this ring via anion···π interactions. The ring rotates to accommodate this proximal nucleophile.

The η^1^-OSO photoisomer in **1** appears to be thermally stable up to about 130 K, whereby it decays into the η^2^-(OS)O photoisomer; this remains stable up to 200–220 K, at which point the sulfur dioxide ligand reverts to its S-bound η^1^-SO_2_ dark-state configuration.

The clean nature of these 100% SO_2_ linkage isomerizations in **1** distinguishes it from other [RuSO_2_] complexes and makes it attractive for prospective applications as an optical actuator.

## Supporting Information Available

Material characterization methods for **1**; photocrystallography results for **1**; and single-crystal Raman spectroscopy for **1** (PDF)
Crystallographic information files (CIFs) of the dark state of **1** used in the paper (CIF)
Crystallographic information files (CIFs) of the light-induced state of **1** used in the paper (CIF)
CIF for the light-induced state of **1** refined using independent diffraction data (CIF)

## Supplementary Material

jp1c02457_si_001.pdf

jp1c02457_si_002.cif

jp1c02457_si_003.cif

jp1c02457_si_004.cif
