# Supplementary material for: Nanooptomechanical Transduction in a Single Crystal with 100% Photoconversion
Source: J Phys Chem C Nanomater Interfaces. 2021 Apr 19;125(16):8907–15. doi: 10.1021/acs.jpcc.1c02457 (PMC8162413; doi:10.1021/acs.jpcc.1c02457)
Supplement: Supplementary file 1 — jp1c02457_si_001.pdf [file jp1c02457_si_001.pdf]

**Supporting Information for:**

# Nanooptomechanical Transduction in a Single Crystal with 100% Photoconversion

Jacqueline M. Cole,[a,b,c,d*] David J. Gosztola,[d] Jose de J. Velazquez-Garcia [a]

[a] Cavendish Laboratory, Department of Physics, University of Cambridge, J. J. Thomson Avenue, Cambridge, CB3 0HE, UK.

[b] ISIS Neutron and Muon Source, STFC Rutherford Appleton Laboratory, Harwell Science and Innovation Campus, Didcot, OX11 0QX, UK.

[c] Department of Chemical Engineering and Biotechnology, University of Cambridge, West Cambridge Site, Philippa Fawcett Drive, Cambridge, CB3 0AS, UK.

[d] Argonne National Laboratory, 9700 South Cass Avenue, Lemont, Illinois 60439, United States

* Author for correspondence (J. M. Cole): jmc61@cam.ac.uk

## Table of Contents

S1 – Materials Characterization Methods for **1** ........................................................S2-S3

S2 – Photocrystallography Results for **1** ........................................................S4-S8

S3 – Single-crystal Raman Spectroscopy and microscopy for **1** ..............................S9-S12

S4 – References ........................................................S13

S5 – Crystallographic information files of the dark- and light-induced state of **1** used in the paper ........................................................(*separate files*)

S5 – Crystallographic information file for the light-induced state of **1** refined using independent diffraction data........................................................(*separate file*)

## S1 - Materials Characterization Methods for 1

**Dark and Photo-induced *In-Situ* Single-Crystal X-ray Diffraction of 1.** The crystal structures of the dark- and light-induced states of **1** were determined using a single-crystal x-ray diffractometer at Argonne National Laboratory, IL, USA. A 0.28 x 0.15 x 0.05 $mm^3$ single crystal of **1** was mounted onto a three-circle Bruker diffractometer equipped with a monochromatic x-ray source (Mo $K\alpha$, $\lambda$ = 0.71073 Å), an Apex CCD detector, and an Oxford Cryosystems open-flow $N_2$ cryostream which maintained the temperature of the crystal at 100 K. A series of data frames were acquired over multiple $\varphi$ and $\omega$ scans of crystal orientations, collected in 0.5° increments each with 20 s exposure time, while maintaining a 50 mm sample-to-detector distance. Data were reduced using SAINT v7.66 software, affording a total of 30233 or 28827 reflections for the dark- or light-induced data collections, respectively. Data for the dark-state crystal structure were first obtained. The crystal was then maintained at 100 K on the diffractometer and held static while 505 nm light was shone onto its thinnest face for 2 h, using a Thorlabs M505L3 light emitting diode (LED) whose head power output was 1000 mA constant current and 3.3 V forward voltage. The crystal was then illuminated for an additional 15 mins at three rotated $\varphi$ orientations, 90°, 180° and 270° from its thinnest face, *i.e.* the crystal was photo-stimulated for 2 hr 45 mins in total for the photo-crystallography experiment. This light was switched off before acquiring data for the light-induced crystal structure. Further experimental details, as well as structure solution and refinement information, are given in the Crystallographic Information Files. A more detailed description of the specialized photocrystallography aspects of these experiments is given elsewhere.[1-5]

**Single-crystal Raman spectroscopy and microscopy of 1.** Raman spectra were recorded using 514.5 nm excitation (Modu-Laser, Stellar) and a Raman microscope (Renishaw, inVia) equipped with a temperature-controlled cryostat stage (Linkam, THMS600) capable of reaching 80 K. Photoisomerization was achieved using the same laser as was used to record the spectra. The crystal sample was mounted onto a sapphire substrate which was placed on the cold finger of the cryostat.

The cryostat was mounted on the XY stage of the optical microscope (Leica DM 2500M) that was part of the Raman system. The sample was illuminated, and Raman scattered light was collected through a 50X long working distance objective (Leica, 0.50 NA, N PlanL). The Raman scattered light was collected in back-reflection geometry using a dichroic filter that reflects the incident laser light and transmits the longer-wavelength Raman scattered light. The system was equipped with an imaging CCD camera allowing for the precise positioning of the excitation spot as well as recording images of the crystal. To minimize crystal orientation effects, the normally linearly polarized laser was circularly polarized using a quarter-wave plate.

The 514.5 nm light source was used to simultaneously photoexcite the crystal and probe it at the same time. A single scan will not yield a fully saturated photoconversion fraction in a single crystal, since a given scan typically lasts only a few minutes. However, Raman spectroscopy is so sensitive to minor structural changes, that even small changes in the vibrational modes of bonds that arise from photoisomerization are detectable. Moreover, the laser needed to be turned down to 0.4% of its total power (ca. 50 $\mu$W bearing in mind the ca. 8% reflective losses due to the 300 $\mu$m thick soft glass cryostat window) to prevent the laser from photodamaging the crystal during the experiment. This suggests that the crystal of **1** had reached its maximum optical absorbance very quickly, within the crystal area that was photoexcited with the laser.

**Single crystal optical absorption spectroscopy and microscopy of 1**. A custom-built micro-spectroscopy system was used to record the absorption spectra of single crystals under a variety of environmental conditions. The system was built around an inverted microscope (Olympus: IX71)

coupled to a 300 mm focal length spectrograph (Princeton Instruments: Acton Series 2300i) and 1320 x 100 channel CCD camera (Princeton Instruments: PIXIS 100BR). A 0.23 x 0.11 x 0.05 $mm^3$ crystal of **1** was mounted on a sapphire disk (9 mm dia., 0.5 mm thick) and fastened with a small amount of viscous perfluoroether oil. The mounted sample was then placed on the cold finger of an optical cryostat (Janis: ST-500-UC) attached to the microscope. The cold finger was drilled through, allowing optical absorption measurements to be made. The crystal was positioned such that only half of the active vertical channels of the CCD camera were used to image a portion of the crystal using a 5 x, 0.13NA objective (Olympus, NeoSPlan); the remaining half of the active detector channels imaged the sapphire substrate. The probe light for optical absorption measurements was provided by the microscope's 100W tungsten-halogen lamp and 0.3 NA condenser optics. A visible bandpass filter (Schott: BG40) and OD 0.9 neutral density filter was placed between the lamp and the condenser to reduce the thermal load on the sample and cryostat.

To induce photoisomerization, the crystal was illuminated with 505nm light from a Thorlabs M505F1 fiber optically coupled light-emitting diode (LED). The light from the LED was collimated and then coupled into the microscope through a side port, focusing it onto the back aperture of the objective, thus filling the field of view and evenly illuminating the entire crystal. The excitation power measured at the objective was typically 635 $\mu$W giving an estimated 28 $\mu W/mm^2$ illuminating the field of view.

Optical absorption spectra were recorded by imaging the crystal on the entrance slit (75 $\mu$m) of the spectrometer and dispersing the light, using a 150 line/mm grating, onto the detector. The image was positioned such that 10 rows of the detector were illuminated with light that passed through the crystal ($I_T$); whereas 10 rows of the detector directly above the crystal recorded light that passed through only the sapphire substrate ($I_0$). Absorption spectra were then calculated as $\log_{10}(I_T/I_0)$.

A more detailed description of this custom instrument set up and its operational pipeline, as provisioned to support photocrystallography, is given elsewhere.[6]

## S2 - Photocrystallography Results for 1

### S2.1 – The need for modeling the bromine as a disordered atom

The bromine atom of **1** was initially crystallographically refined using a libration model, as per the pyridyl carbon atoms which display significant libration. The corresponding anisotropic displacement parameters (ADPs) of the pyridyl ring seem to be subject to a twist. Thereby, the bromine atom seems to pull the nearest carbon atoms out of the ring plane, while the Ru-Npyr coordinative bond tempers this pull as evidenced by the competing alignments of carbon ADPs that lie closer to this bond. Figure S2 displays this twist in **1**.

At first sight, a corresponding libration model for the adjoining bromine atom would seem to make sense. However, all attempts to model the bromine atom with a single ADP were unsatisfactory. An R1 value of 12% provided the best possible figure-of-merit for such a model, which was clearly unsatisfactory, especially when considering that the refined bromine atom in this model was surrounded by a substantial level of residual (unmodeled) electron density.

Having exhausted all possible avenues to improve such a libration model, the bromine atom was modeled via positional disorder. The libration model was otherwise left unchanged, there being a single change in modeling the bromine atom as positional disorder (two independent ADPs) rather than as a single ADP (the libration model). The results were striking: the R1 value reduced from 12% to less than 7% with this single change. The difference electron density maps with bromine modeled as one or two ADPs (Figures S2 and S3, left and right, respectively) reveal the full 3-D impact on the crystal structure determination.

The magnitude and sense of the residual electron density in the crystallographic asymmetric unit of **1** is most evident from Figure S2(left) which shows when bromine was refined as a libration model (a single ADP). This ADP is heavily elongated and yet it is entirely obscured by the massive lobes of positive (green) and negative (red) electron density that remain unmodeled. It is also noteworthy that the charge of this residual electron density is nodal, as evidenced by the alternating green and red lobes. There is little residual electron density elsewhere within the asymmetric unit. The orientation of this residual electron density with respect to the neighboring tosylate ions is best seen within a packing diagram of the crystal lattice when viewed down the a, b and c crystallographic axes (Figure S3, left). There is a particularly marked projection of such density towards the disordered arene ring (the 'rotor ring', as defined within the main manuscript).

Once the bromine atom has been modeled as positional disorder (two independent ADPs), no residual electron density is visible around this atom at all (making the comparison between all plots, using the same isovalue of 2.29 eA-3; see Figures S2 and S3, right). Indeed, the largest amount of residual electron density in this model surrounds the ruthenium core which one would expect given that it is a heavy atom. The two independent ADPs for Br1 and Br1A extend towards the non-disordered and disordered rings of the two tosylate ions, respectively. The ADP of Br1 is heavily elongated towards the non-disordered ring, whose carbon ADPs align in the same direction. Thus, the vibrational forces would seem to be correlated, although Br1 and the ring are not so close that significant interactions can form; in fact, Br1 makes closer interactions with atoms from a neighboring ring rotor (see Figure S1, Table S1). Moreover, the bond lengths in this ring are quite uniform (see Figure 2 in the main manuscript), so it does not have an innate electrostatic propensity to interact. The ADP of Br1A is much more spherical in form, and projects directly towards its neighboring rotor ring. Its more spherical form would suggest that it makes close and defined interactions with this ring; it is also held in place quite isotropically by a range of non-

bonded contacts as shown in Figure S1 and Table S1. The rotor ring seems to rotate in order to best accommodate Br1A, thereby offering some energy-stabilization to the crystal lattice environment.

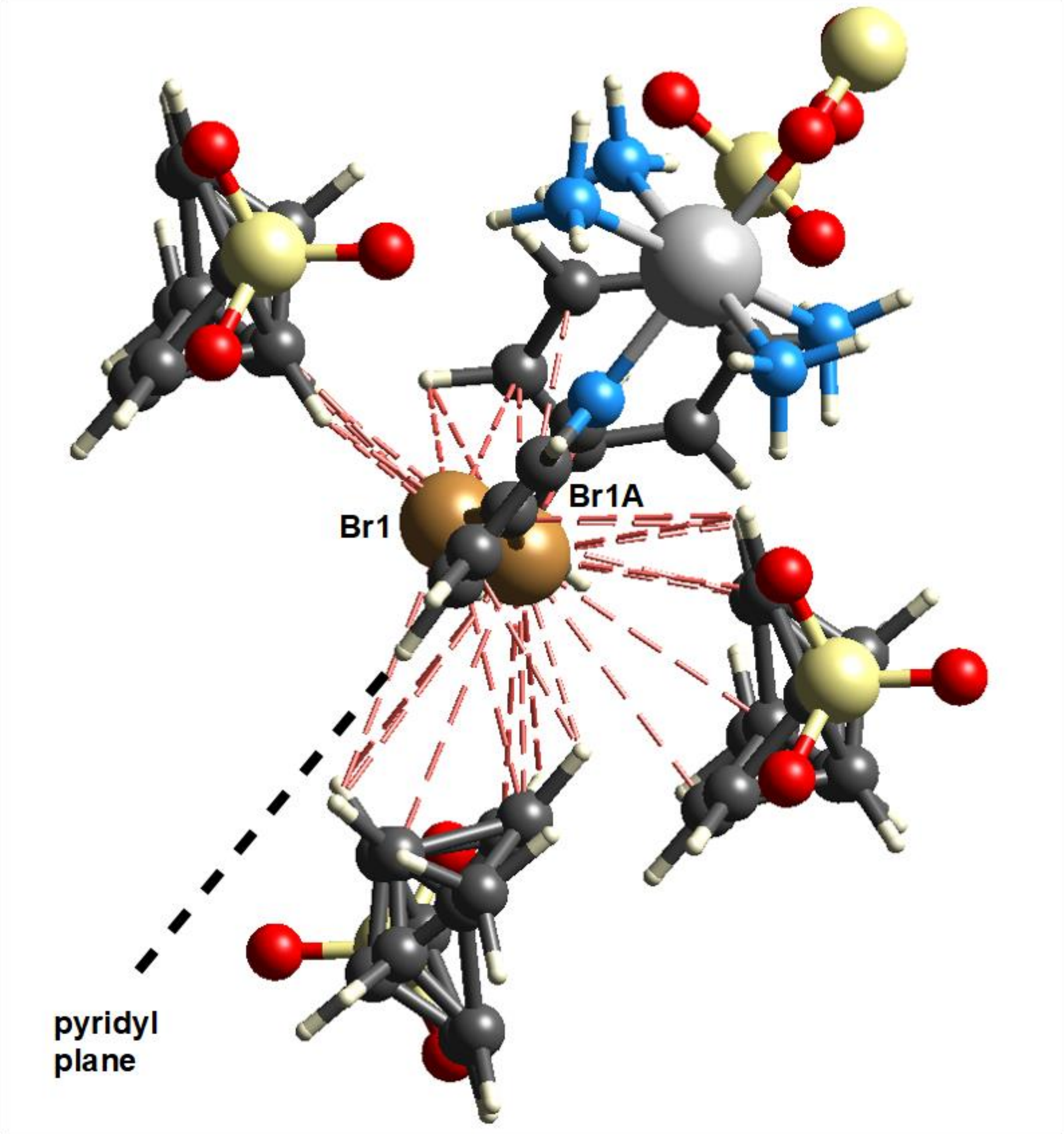


**Figure S1 –** Br...arene interactions in **1**. This plot was rendered using CrystalExplorer17.[6]

**Table S1** - *Br···arene interaction lengths in **1** taken from the structure determination shown in the manuscript. Only the rotor ring possesses such interactions with a separation less than 3 Å.*

| **Br···arene interaction** | **Br···arene interaction length /** *Å* |
|---|---|
| *Br1···H21B-C21* | 2.244 |
| *Br1···H22B-C22* | 2.606 |
| *Br1A···H22A-C22* | 2.508 |
| *Br1A···H24B-C24* | 2.860 |
| *Br1A···H25B-C25* | 3.032 |

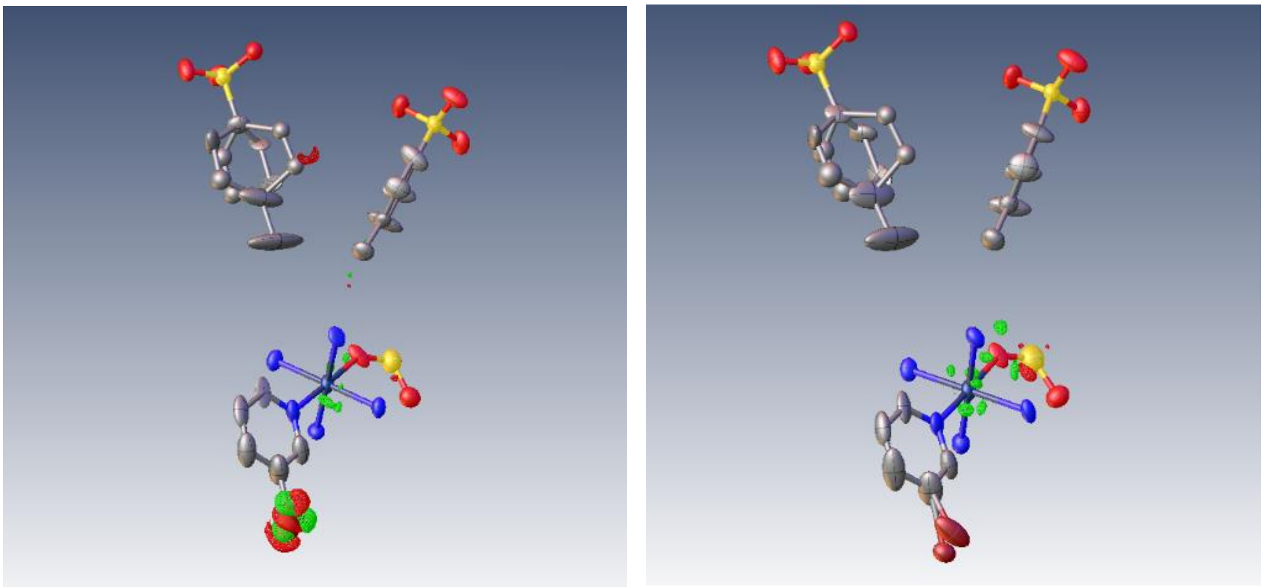

**Figure S2 -** 3-D Difference electron density maps of the crystallographic asymmetric unit of **1**. Green and red wire frame represents areas of positive and negative electron density, respectively, using an isovalue of 2.29 eÅ$^{-3}$. These plots were rendered using the Olex 2 software package.[7]

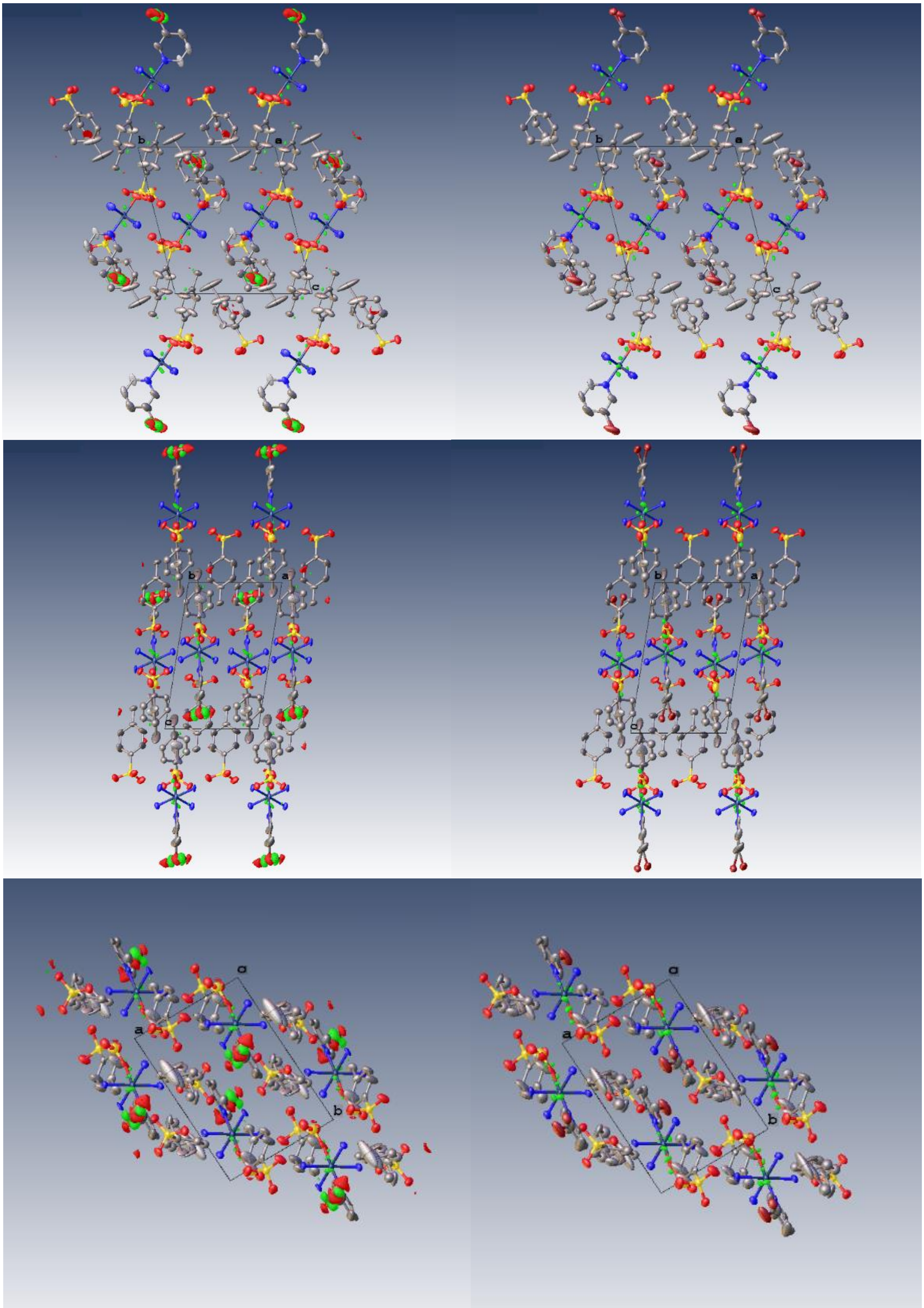


**Figure S3** – 3-D Difference electron density maps of **1**. Green and red wire frame represents areas of positive and negative electron density, respectively, using an isovalue of 2.29 eÅ$^{-3}$, displayed looking down the (top) a (middle) b (bottom) c crystallographic axis. Visualized using Olex2.[7]

**S2.2 - Duplicate crystal structure determination of the light-induced structure of 1 with independent diffraction data to demonstrate the verity of our mechanistic structural findings**

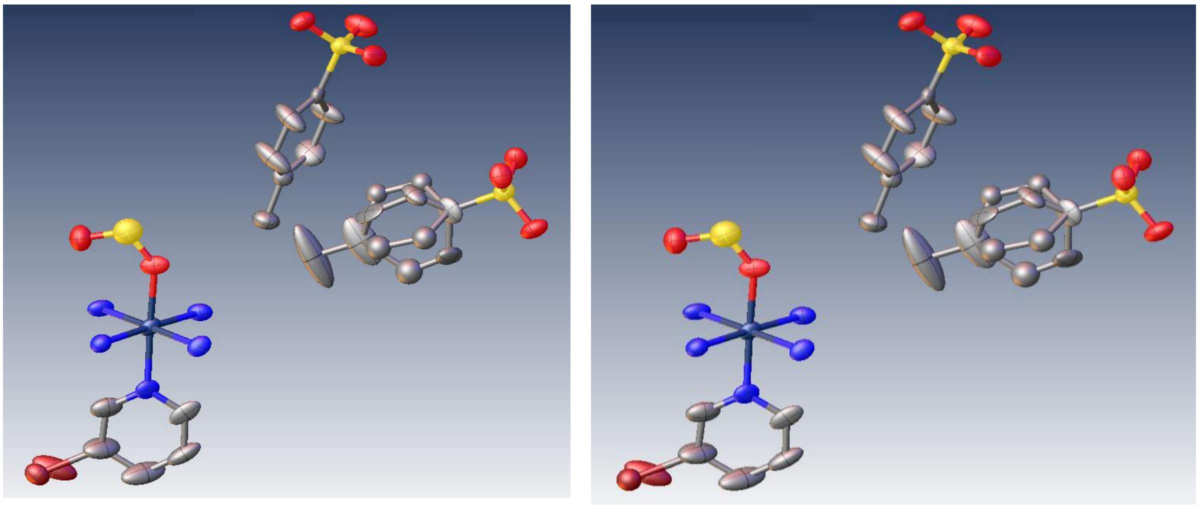

(a) (b)

**Figure S4** - Comparison of light-induced crystal structures of **1**: (a) the structure presented in the manuscript; (b) a duplicate crystal structure determination of 1 from a different data acquisition. Atoms are shown in the following colors: S = yellow; C = grey; O = red; N = blue; Br = scarlet. Hydrogen atoms are omitted for the purposes of clarity. Visualized using Olex2.[7]

**S3 - Single-crystal Raman Spectroscopy Results for 1**

The single-crystal Raman spectroscopy results complemented the photocrystallography results in that they evidenced a range of vibrational modes that are due to local photostructural changes in **1**. Resonance Raman effects are prevalent in ruthenium ions [8] and the high intensities seen in this experiment are consistent with the ruthenium cation in **1** exhibiting such phenomena. Accordingly, Raman shifts in **1** correspond entirely to the ruthenium-based cation of **1**, while any contributions from tosylate ions are too weak to observe. A summary of the peak assignments to the Raman spectral shifts for the light-induced structure of **1** is given in Table S2. These assignments were made from the multi-temperature Raman spectra that are shown in Figure S5. The full temperature range of Raman spectra measurements are displayed in a stacked plot in Figure S6.

***Table S2*** *– A summary of vibrational modes in* ***1*** *classified from Raman shifts in the dark-state spectrum at 300 K (dark) and that once exposed to 514.5 nm light at 90 K (light). w, m and s indicate weak, medium and strong peak intensities.*

| **Raman shift / $cm^{-1}$, $\eta^1$-$SO_2$ isomer, 300 K** | **Raman shift / $cm^{-1}$, $\eta^1$-OSO isomer, 90 K** | **Raman shift / $cm^{-1}$, $\eta^2$-(OS)O isomer, 140 K** | **Classification of vibrational mode** | **References to related classifications** |
|---|---|---|---|---|
| 347 (s) | 334 (s) | 357/373 (s) | Ru-S, Ru-O or Ru-(OS) stretching mode | [9][6] |
| 554 (m) | 541 (s) | 541 (s) | Deformation mode of $SO_2$ | [10] |
| 1123 (s) | 1129 (m) | 1129 (m) | $SO_2$ symmetric stretching mode | [6][10] |
| 1289 (m) | 1283 (vw) | 1292 (vw) | $SO_2$ asymmetric stretching mode | [6][10] |
| 3074 (w) | 3054 (w) | 3058 (w) | 3-Bromopyridine CH mode | |
| 3184 (w) | 3199 (m) | 3197 (vw) | $NH_3$ stretching modes | [6][11] |

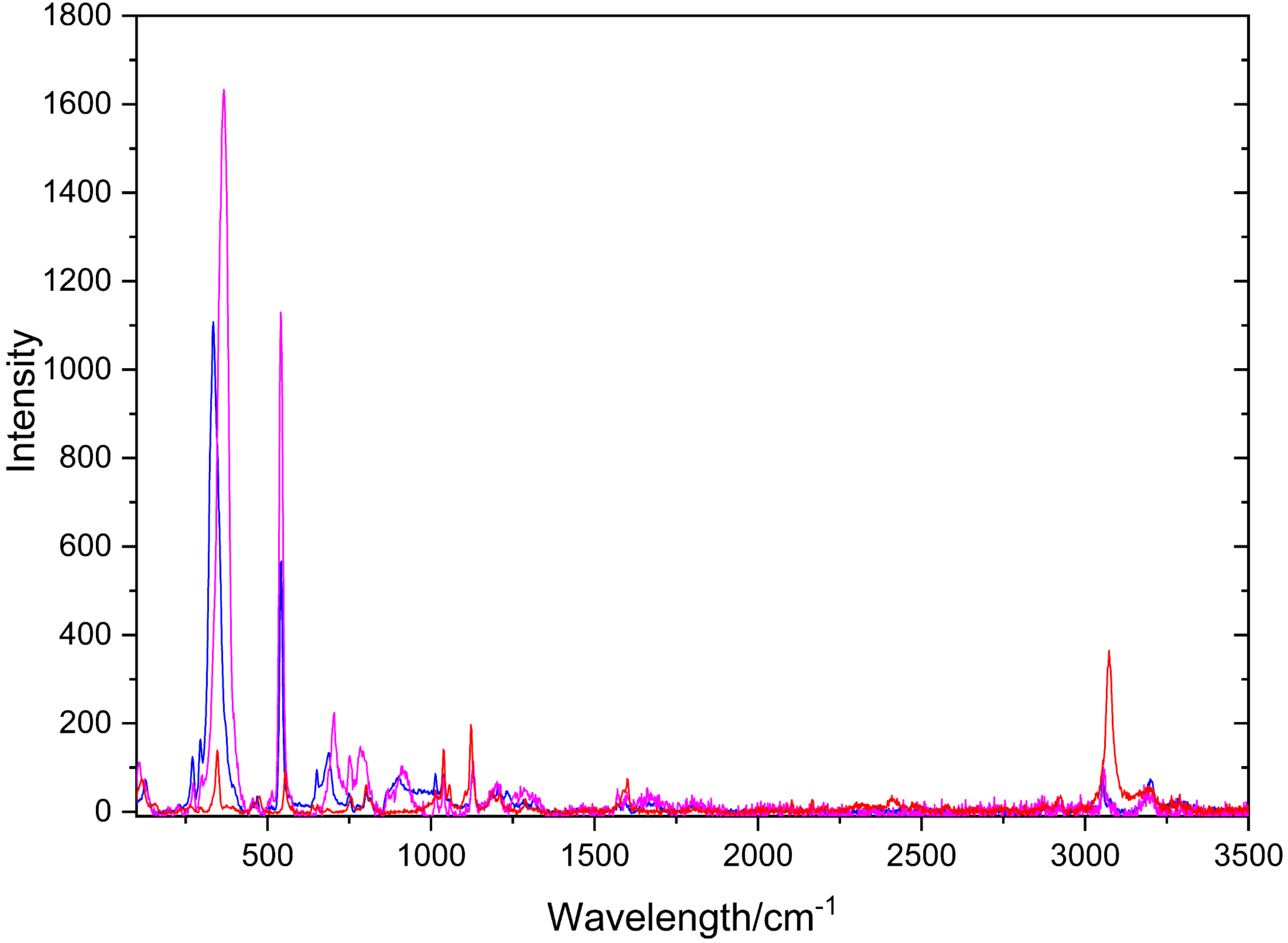


**Figure S5** – Single-crystal Raman spectra of **1** acquired at 90 K (blue), 140 K (magenta) and 300 K (red).

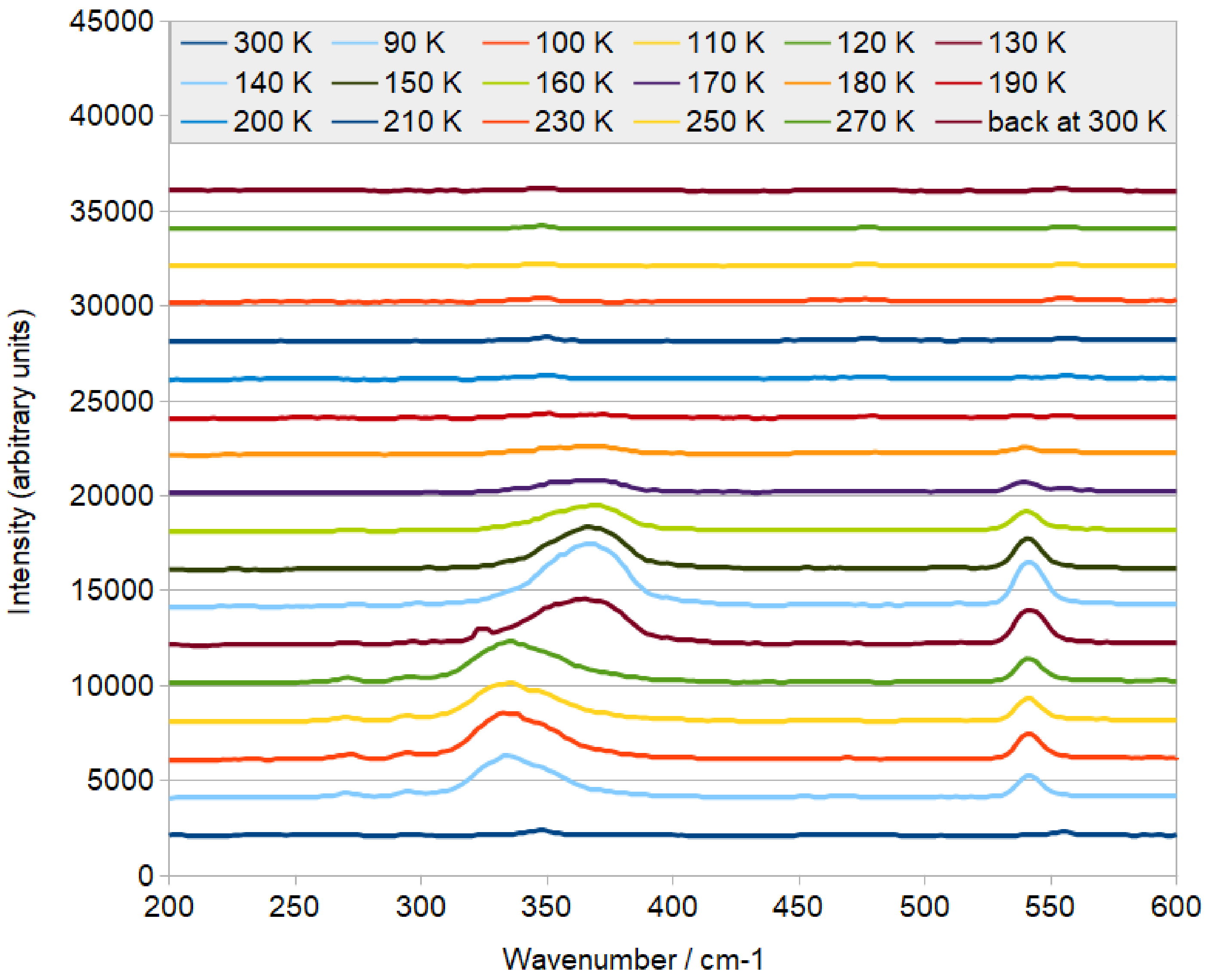


**Figure S6** – Multi-temperature single-crystal Raman spectra of **1** (stacked plot)

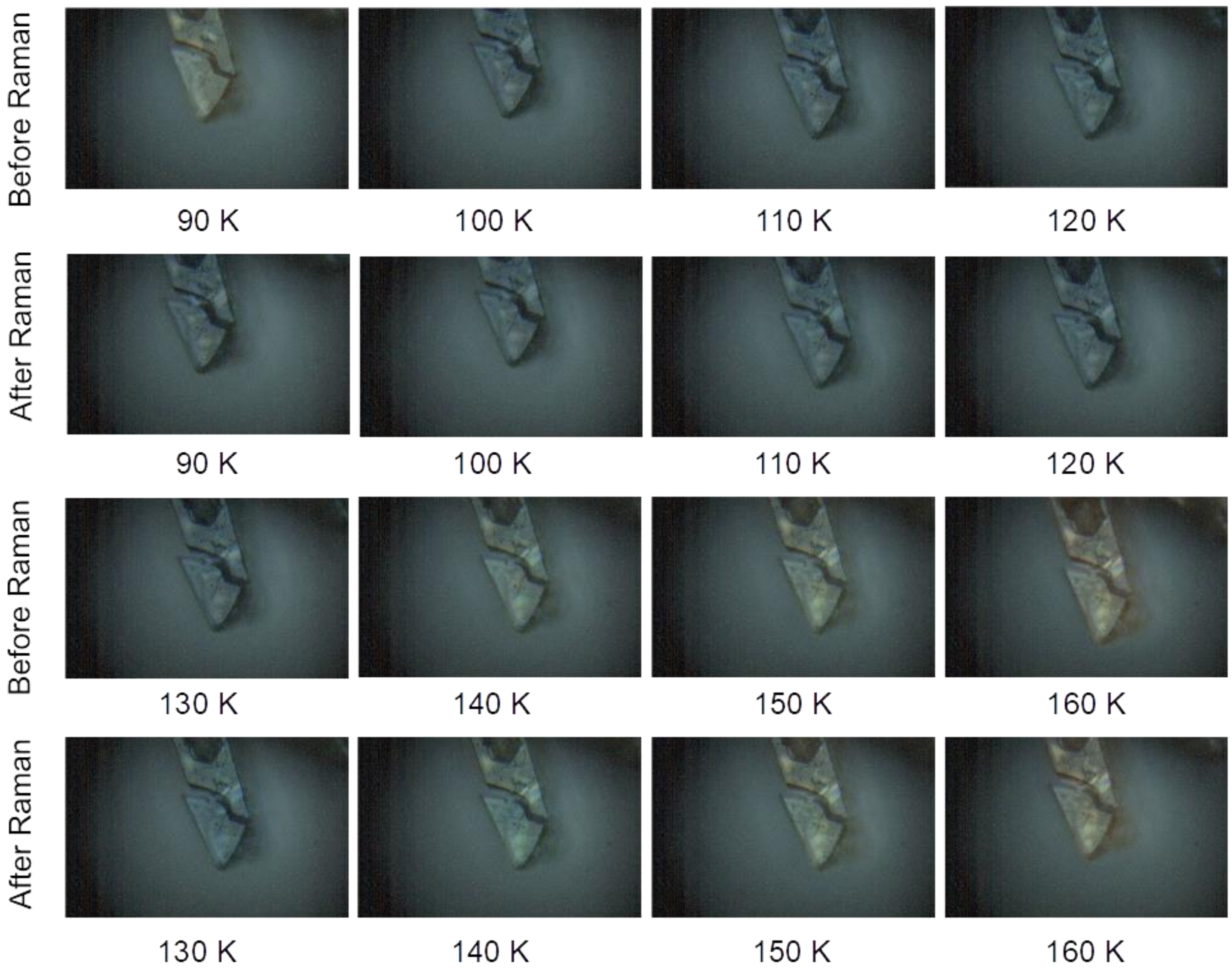


**Figure S7 -**Multi-temperature images of a single crystal of **1** from a Raman microscope, revealing photochromic effects that onset at 140 K.

**S4 – References**

[1] P. Coppens, D. V. Fomitchev, M. D. Carducci and K. Culp, *J. Chem. Soc., Dalton Trans*., 1998, 6, 865-872.
[2] Cole, J. M. *Chem. Soc. Rev*., 2004, 33, 501-513.
[3] Cole, J. M. *Acta Crystallogr., Sect. A: Found. Crystallogr*., 2008, 64, 259-271.
[4] Cole, J. M. *Analyst*, 2011, 136, 448-455.
[5] Cole, J. M. *Z. Kristallogr.* 2008, 223, 363– 369,
[6] Cole, J. M.; Gosztola, D. J.; Velazquez-Garcia, J. d. J.; Chen, Y-S. *J. Phys. Chem. C* **2020**, 124, 51, 28230–28243.
[7] Turner, M. J.; McKinnon, J. J.; Wolff, S. K.; Grimwood, D. J.; Spackman, P. R.; Jayatilaka, D.; Spackman, M. A. CrystalExplorer17 (2017). University of Western Australia. https://hirshfeldsurface.net.
[8] Dolomanov, O.V.; Bourhis, L.J.; Gildea, R.J.; Howard, J.A.K.; Puschmann, H., OLEX2: A complete structure solution, refinement and analysis program (2009). *J. Appl. Cryst*., 42, 339-341.
[9] Lauter, M.; Breitinger, D. K.; Breiter, R.; Mink, J.; Bencze, E. *J. Mol. Struct.* **2001**, *563-564*, 383-388.
[10] Breitinger, D. K.; Breiter, R. *J. Mol. Struct*. 1995, 349, 45-48.
[11] Vogt, L. H.; Katz, J. L.; Wiberley, S. E. *Inorg. Chem.* 1965, 4, 1157-1163.
